# Sperm aneuploidy and DNA fragmentation in unexplained recurrent pregnancy loss: a multicenter case-control study

**DOI:** 10.1186/s12610-018-0070-6

**Published:** 2018-04-02

**Authors:** Camille Esquerré-Lamare, Marie Walschaerts, Lucie Chansel Debordeaux, Jessika Moreau, Florence Bretelle, François Isus, Gilles Karsenty, Laetitia Monteil, Jeanne Perrin, Aline Papaxanthos-Roche, Louis Bujan

**Affiliations:** 1 Research Group on Human Fertility EA 3694, University Paul Sabatier Toulouse III, 330 av Grande Bretagne, 31059 Toulouse, France; 2 CECOS Groupe d’Activité de Médecine de la Reproduction, CHU Toulouse, Toulouse, France; 3 CECOS Service Biologie de la Reproduction, CHU Bordeaux, Bordeaux, France; 4 Service de Gynécologie-Obstétrique, AP-HM Hôpital Nord Marseille, Marseille, France; 5 Andrologie, Groupe d’Activité de Médecine de la Reproduction, CHU Toulouse, Toulouse, France; 6 Service d’Urologie et Transplantation Rénale, AP-HM La Conception, Marseille, France; 7 Service de Génétique Médicale, CHU Toulouse, Toulouse, France; 8 CECOS Laboratoire de Biologie de la Reproduction, AP-HM La Conception, Marseille, France; 9 Mediterranean Institute for Biodiversity and Ecology (IMBE), Aix-Marseille Université, CNRS, IRD, Avignon University, Marseille, France

**Keywords:** Unexplained recurrent pregnancy loss, Fluorescence in situ hybridization, Sperm aneuploidy, Sperm DNA fragmentation, Fausses couches à répétition inexpliquées; aneuploïdie du spermatozoïde; fragmentation de l’ADN du sperme; facteur masculin.

## Abstract

**Background:**

Recurrent pregnancy loss (RPL) is defined as the loss of at least three pregnancies in the first trimester. Although the most common cause is embryo aneuploidy, and despite female checkup and couple karyotyping, in about 50% of cases RPL remain unexplained. Male implication has little been investigated and results are discordant. In this context, we conducted a multi-center prospective case-control study to investigate male gamete implication in unexplained RPL.

**Methods:**

A total of 33 cases and 27 controls were included from three university hospitals. We investigated environmental and family factors with a detailed questionnaire and andrological examination, sperm characteristics, sperm DNA/chromatin status using the sperm chromatin structure assay (SCSA) and terminal deoxynucleotidyl transferase dUTP nick end labeling (TUNEL) and sperm aneuploidy using fluorescence in situ hybridization (FISH). The Mann-Whitney test and the Wilcoxon or Fisher exact tests were used. A non-parametric Spearman correlation was performed in order to analyze the relationship between various sperm parameters and FISH and sperm DNA fragmentation results.

**Results:**

We found significant differences between cases and controls in time to conceive, body mass index (BMI), family history of infertility and living environment. In cases, total sperm motility and the percentage of morphologically normal spermatozoa were significantly decreased. No difference was found between cases and controls in sperm DNA fragmentation or chromatin integrity. In cases, spermatozoa with aneuploidy, hyperhaploidy and chromosome 18 disomy were significantly increased.

**Conclusions:**

This prospective case-control study is one of the largest to examine environmental factors, sperm characteristics, sperm DNA fragmentation and chromatin, and chromosome anomalies in spermatozoa in relation to unexplained recurrent pregnancy loss. The originality of our study lies in the comprehensive andrological examination and search for risk factors and fertility history. Further studies are needed to confirm the links between unexplained RPL and a male family history of infertility or miscarriages. The increased sperm aneuploidy observed in unexplained RPL supports a male etiology. These data pave the way for further studies to demonstrate the value of preimplantation genetic screening in men with increased sperm aneuploidy whose partners experience unexplained RPL.

## Résumé

**Contexte:**

Les fausses couches à répétition (FCR) sont définies lorsqu’au moins trois fausses couches ont eu lieu au cours du premier trimestre. Bien que la cause la plus fréquente soit l’aneuploïdie embryonnaire, et malgré un bilan chez la femme et un caryotype du couple, dans environ 50% des cas, les FCR restent inexpliquées. L’implication masculine a été peu étudiée et les résultats restent discordants. Ainsi, nous avons réalisé une étude cas-témoins prospective et multicentrique afin d’investiguer l’implication du gamète mâle dans les FCR inexpliquées.

**Méthodes:**

Un total de 33 cas et de 27 témoins ont été inclus recrutés au sein de trois hôpitaux universitaires. Nous avons étudié les facteurs environnementaux et familiaux à partir d’un questionnaire détaillé ainsi que les données de l’examen andrologique, les caractéristiques du sperme, la fragmentation de l’ADN et la chromatine du spermatozoïde en utilisant le sperm chromatine structure assay (SCSA) et le test du TUNEL, ainsi que l’aneuploïdie des spermatozoïdes grâce à la méthode d’hybridation in situ de sonde chromosomique (FISH). Le test de Mann-Whitney et les tests exacts de Wilcoxon ou de Fisher ont été utilisés. Une corrélation de Spearman non-paramétrique a été réalisée afin d’analyser la relation entre les divers paramètres de sperme et les résultats de fragmentation d’ADN du sperme et les résultats de la FISH.

**Résultats:**

Nous avons trouvé des différences significatives entre les cas et les témoins pour le délai de conception, l’indice de masse corporelle (IMC), les antécédents familiaux d’infertilité et le milieu de vie. Chez les cas, la mobilité totale des spermatozoïdes et le pourcentage de spermatozoïdes normaux étaient significativement diminués. Aucune différence n’a été trouvée entre les cas et les témoins concernant la fragmentation de l’ADN des spermatozoïdes ou l’intégrité de la chromatine. Chez les cas, la fréquence des spermatozoïdes présentant une aneuploïdie, une hyperhaploïdie et une disomie du chromosome 18 étaient significativement augmentée.

**Conclusions:**

Cette étude cas-témoins prospective est. l’une des plus importantes ayant investigué à la fois les facteurs environnementaux, les caractéristiques des spermatozoïdes, la fragmentation et la chromatine de l’ADN des spermatozoïdes et les anomalies chromosomiques des spermatozoïdes en rapport avec les fausses couches à répétition inexpliquée. L’originalité de notre étude réside dans l’examen andrologique complet et la recherche des facteurs de risque et des antécédents reproductifs. D’autres études sont nécessaires pour confirmer les liens entre les FCR inexpliquées et les antécédents familiaux masculins d’infertilité ou de fausses couches. L’augmentation de l’aneuploïdie des spermatozoïdes observée chez les cas présentant des FCR inexpliquées plaide en faveur d’une étiologie masculine. Ces données ouvrent la voie à d’autres études pour démontrer l’utilité d’un dépistage génétique préimplantatoire chez les hommes présentant une augmentation de l’aneuploïdie des spermatozoïdes dont les partenaires présentent des FCR inexpliquées.

## Background

Early pregnancy loss is defined as a miscarriage occurring before 12 weeks of gestation. The most common cause of these losses is embryo aneuploidy. Risk factors include uterine anomalies, metabolic and hormonal disorders, infection, chronic endometritis, autoimmune anomalies, thrombophilia and parental chromosomal translocations [1]. However, around 50% of miscarriages remain unexplained. Recurrent pregnancy loss (RPL) is defined as three or more consecutive early pregnancy losses in the first trimester of gestation. Studies on male factors involved in these pregnancy losses are recent, and several factors have been found, notably related to semen characteristics. The total number of motile spermatozoa has also been found to be significantly decreased in RPL compared with fertile men [2, 3] and morphological sperm alterations are more numerous [4–11].

It is noteworthy that sperm DNA fragmentation is involved in the late paternal effect on human embryo development in cases of repeated failures of assisted reproduction techniques (ART), with a presumed effect on implantation failure [12], post-implantation embryo development and increased pregnancy loss [13]. Gil-Villa et al.. found an increased DNA fragmentation index (DFI) in 9 out of 17 patients with RPL after spontaneous pregnancy [14]. Kumar et al. found that DFI values differed significantly between RPL cases and controls [15]. Using the TUNEL technique, Brahem et al. found that men in the RPL group had significantly higher values of DNA fragmentation than the control group [6]. Furthermore, Zidi-Jrah et al.. showed similar results since 45% of men in the RPL group had an abnormal TUNEL value [7].

Abnormal sperm aneuploidy has been investigated in a few studies as a possible cause of RPL. In men whose partners experienced RPL, Bernardini et al found significantly higher values for chromosome 1 and 8 disomy and total aneuploidy [3], as did Collodel et al. The latter authors also found increased diploidy, disomy and total aneuploidy for chromosomes 18, X and Y [5]. Two studies on chromosomes 18, 13, 21 X and Y found an increased aneuploidy rate in the case group compared with normospermic men [16], with the general population and with fertile men [11]. A recent study which scanned all the chromosomes in sperm from 11 male partners of couples experiencing RPL found increased aneuploidy for chromosomes 16, 2, 1, 21, 4 and 6 [17]. Lastly, Zidi-Jrah et al. observed that all men in the RPL group (*n* = 22) presented a significant increase in sex chromosome disomy and nullisomy as well as a significantly higher rate of diploidy compared with the control group (*n* = 20) [7].

In the light of the few studies on sperm fragmentation and RPL and of the sparse research focusing on sperm aneuploidy and RPL, our prospective case-control study focused on male implication in RPL by studying all three parameters (sperm DNA fragmentation, sperm chromatin condensation status and sperm aneuploidy) in 33 men whose partners experienced unexplained RPL (cases) and by comparing results with those of 27 men of proven fertility (control group). We also sought risk factors with an extensive questionnaire and thorough andrological examination.

## Methods

### Study participants

The study was a prospective case/control study. Patients (cases) and controls were recruited in the university hospitals of three French cities (Bordeaux, Marseille and Toulouse).

Cases were recruited when they came to the fertility centers for management of unexplained recurrent pregnancy loss (URPL).

Controls were recruited in the maternity departments of the same hospitals. They had proven fertility (full-term pregnancy with live birth, baby aged less than a year old when entering the study). They also had no history of ART treatment or RPL (i.e. no more than two recurrent miscarriages before 12 weeks of gestation).

### Inclusion

Men were included in the case group if their couple had experienced more than two recurrent miscarriages before 12 weeks of gestation, with no female causes identified after a classic RPL check-up: anatomical causes (investigated by hysterosalpingography, hysteroscopy or 2D/3D ultrasound); endocrinological causes (measurement of TSH, anti-thyroid antibodies and FSH); autoimmune causes (measurement of anticardiolipin antibodies and lupus anticoagulant); thrombophilia (measurement of homocysteine, factor V Leiden, prothrombin and C-reactive protein). Infectious causes were not investigated unless the woman had chronic endometriosis, cervicitis or immunodepression. All pregnancies occurred naturally. The female partners were under 38 years old. Blood karyotypes were normal in both men and women. The men were healthy, with no identifiable cause of infertility, no family disease and no endocrine or immunological disorders. The controls included were age-matched ±3 years.

At inclusion, controls and cases were asked about fertility histories within the family and outside the couple, and the fertility history of their own couple (pregnancies, miscarriages, time to conceive). Surgical and medical histories, history of infections, professional exposure, and andrological conditions such as urinary infection, varicoceles, cryptorchidism and family histories of these conditions were investigated. Environmental factors were investigated by evaluating alcohol, caffeine and tobacco consumption, living environment and lifestyle. The male partner underwent clinical examination and a detailed questionnaire was completed on the female partner.

All participants were recruited on a voluntary basis and gave informed consent. This study was supported by a grant from the French Ministry of Health (PHRC N°11, 198 08, 2011, PARTHOM project). The sponsor had no role in the study. The project was approved by the institutional ethics review board (Comité de Protection des Personnes Sud Ouest et Outre-Mer II). This work was sponsored by the Universitary Hospital of Toulouse for regulatory and ethic submission.

### Semen analysis

Sperm samples were collected by masturbation after 3–6 days of sexual abstinence. Semen was allowed to liquefy for 30 min at 37 °C before analysis. Conventional semen analysis was performed according to WHO guidelines with similar methodology in the three laboratories. Sperm analysis is subject to external quality control in all centers. Ejaculate volume (vol, ml), sperm count (SC, 10^6^ spermatozoa/ml), total sperm count (TSC = vol x SC, 10^6^ spermatozoa/ejaculate), motility (percentage of rapidly progressive or slowly progressive spermatozoa), total motile sperm count (TMSC = TSC x motility, 10^6^ spermatozoa/ejaculate) and vitality were assessed.

Sperm cell smears were prepared in each center for sperm morphology analysis. Morphology was studied according to David’s classification modified by Jouannet [18, 19]. We calculated the multiple anomalies index (MAI) which was the number of anomalies per abnormal spermatozoon. In order to reduce interoperator variability, sperm morphology analysis was performed blindly by a single technician in the Toulouse center.

The remaining semen sample was mixed with a cryoprotectant, frozen in straws within 1 h of collection and stored in liquid nitrogen according to the standard procedures used for sperm banking in the laboratories. All samples were stored in the Germethèque Biobank (BB-0033-00081, France) until further analysis centralized in Toulouse.

### Sperm chromatin and DNA fragmentation exploration

Sperm chromatin condensation was evaluated in each center by aniline blue staining of semen samples fixed with 3% glutaraldehyde according to a previously published method [20].

Sperm DNA fragmentation index (DFI) and high DNA stainability (HDS) were measured by conventional sperm chromatin structure assay (SCSA) described by Evenson and Jost and routinely used in our laboratory as previously published [20].

DNA strand breaks were measured using the terminal deoxynucleotidyl transferase dUTP nick end labeling (TUNEL) assay according to a previously published method [20].

SCSA and TUNEL assay were centralized in the Toulouse laboratory. 5000 and 10,000 cells were analyzed by SCSA and TUNEL, respectively, by fluorescence-activated cell sorting (FACS) on a FC500 cytometer (Beckman Coulter Inc, Fullerton, CA, USA).

### Sperm fluorescence in situ hybridization (FISH)

In the Toulouse center, cells were thawed and then washed twice with 5 ml PBS and centrifugation at 630 g. Samples were then fixed with fixation solution (acetic acid and methyl alcohol) for 30 min at 4 °C. After centrifugation at 1500 g, the supernatant was discarded and the pellet resuspended. 10 μl were dropped on a slide and cell density was verified by microscopy and adjusted accordingly. Slides were incubated for a minimum of 2 h at − 20 °C. DNA was decondensed by incubating slides in 1 M NaoH for 1 min, washed twice in SSC, then dehydrated in 70%, 90% and 100% ethyl alcohol baths (2 min each). Each slide was then incubated overnight with the different probes at 37 °C (Vysis probes (Abbott), CEP X spectrum green, CEP Y spectrum orange and CEP 18 spectrum aqua). After a 2 min wash in 2SSC 0.4% NP40 at 73 °C followed by a 1 min wash in 2SSC 03% NP40, slides were incubated with 1/2000e Hoechst for 3 min and washed for 3 min in PBS. Slides were finally mounted with Antifade mounting medium (Promega, Germany) and stocked at − 20 °C until read. Slides were analyzed under a Leica DM 6000 B microscope system. At least 5000 cells were read by a single reader for each slide.

### Statistical analysis

Quantitative data were presented as median and interquartile range [q1–q3] due to the number of participants and as boxplots for graphic representation.

Standard non-parametric tests were used to compare cases and controls. For all quantitative data, the Mann-Whitney test was used. For qualitative data, differences between subgroups were tested with the Wilcoxon test and Fisher exact test. A non-parametric Spearman correlation was performed to analyze the relationships between various sperm parameters and FISH results, as well as sperm DNA fragmentation results and FISH results. All univariate and multivariate analyses were performed using SAS 9.3 software and the significance level was defined as 5%.

The datasets used and analysed during the current study are available from the corresponding author on reasonable request.

## Results

### Population (Table 1)

A total of 33 volunteers were included in the URPL group (case group) and 27 in the control group. As expected, the number of miscarriages was significantly higher in the case group (*P* < .001). The number of pregnancies was also significantly higher among cases, 5 [3-6] compared with 2 [1-6] in controls (*P* < .001). Time to pregnancy was significantly higher in the case group (3 [1-6] months versus 2 [1-6] months in controls, *P =* .046).

**Table 1:** Medical, Family and Environmental Histories of the 33 Cases and 27 Controls

	Cases		Controls	
	median	[q1–q3]	median	[q1–q3]
Couple’s fertility history				
Pregnancy (conception)*	5	[3-6]	2	[1-6]
Live birth*	0	[0–1]	1	[1-4]
Miscarriage*	3	[3-6]	0	[0–2]
Time to conceive for the first conception (months)	3	[0–18]	2	[0–18]
Time between the end of the first conception and the beginning of the second attempt at conception (months)	6	[0–64]	16	[1–80]
Andrological, family and environmental histories, and lifestyle habits of men				
Age (years)	34	[30–36]	33	[29–35]
Body mass index (BMI)*	25	[23-27]	24	[22-25]
	n	(%)	n	(%)
History of infections	3	(9)	6	(22)
Cryptorchidism	2	(6)	1	(4)
Varicoceles	2	(6)	3	(11)
Normal vas deferens	33	(100)	27	(100)
Abnormal epididymal position	0	(0)	1	(4)
Puberty				
before 12 years old	2	(6)	1	(4)
after 12 years old	29	(94)	26	(96)
Family history of infertility*	16	(53)	6	(24)
Family history of miscarriage*	9	(36)	2	(9)
Family history of cancer	13	(43)	8	(33)
Tobacco status				
< 10/day	4	(12)	4	(15)
≥ 10/day	7	(21)	7	(26)
Drug consumption	6	(18)	6	(22)
Alcohol consumption				
occasional	29	(88)	22	(81)
< 1 L/day	1	(3)	4	(15)
Environment				
*: large urban	12	(36)	19	(70)
small urban	13	(39)	5	(19)
rural	8	(24)	3	(11)
Environmental/professional exposure	12	(36)	6	(22)

*P < .05 between cases and controls

The median age of the men did not differ between the URPL and control groups (34 [30–36] and 33 [29–35] years, respectively) but male body mass index (BMI) was significantly higher in the URPL group than in controls (25 [23-25] versus 24 [22-25], *P* = .025). Regarding andrological histories and clinical examination, no differences between cases and controls were found. Cases were more likely to have a family history of infertility (53% vs 24% of controls, *P* = .031) and miscarriages were more frequent (36% vs 9% of controls, *P* = .041).

**Fig. 1:**
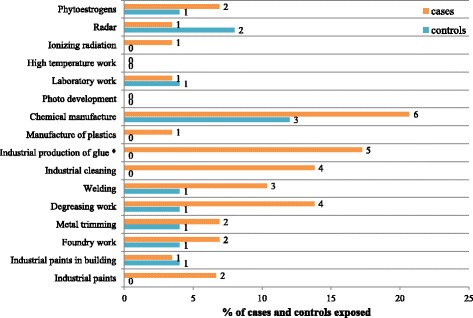
Professional exposure of men with unexplained RPL and controls. Numbers in the figure are the number of men. *P < .05 between cases and controls

There also was a significant difference in the living environment. Cases mainly lived in small urban areas whereas controls preferentially lived in large urban areas (*P* = .032). The results concerning professional exposure are presented in Fig. 1.

The age of the female partner did not significantly differ between the two groups, with a median age of 31 [30–35] years in the URPL group vs 30 [27-33] years in controls. No difference was found for female BMI (data not shown).

### Semen characteristics

As shown in Table 2, duration of abstinence was significantly shorter in cases than in controls (4 [3-5] days vs 4.5 [3.5–6.5] days, *P* = .046). Significant differences in semen characteristics between men in the URPL group and controls were found for TMSC (75.2 × 10^6^ [55.6–124.8] vs 142.1 × 10^6^ [67.7–287.7], *P* = .035) and for percentage of normal spermatozoa (29% [17–42] vs 38% [26–44], *P* = .043). After adjusting for duration of abstinence and male age, no other semen characteristics were significantly different between cases and controls.

**Table 2:** Sperm Characteristics and Semen Morphology of the 33 Cases and 27 Controls

	Cases		Controls	
	median	[q1–q3]	median	[q1–q3]
Abstinence duration (days)*	4	[3–5]	4.5	[3.5–6.5]
Volume (mL)	3.2	[2.3–4.1]	3.1	[2.4–4.3]
Sperm count (× 10^6^/mL)	65	[35.6–90]	79	[53–156]
Total sperm count (×10^6^ per ejaculate)	180	[123.5–369]	338.4	[134.3–540]
Motility (%)	45	[35–55]	50	[30–65]
Vitality (%)	72	[65–81]	70	[64–84]
Total motile sperm count (×10^6^)*	75.2	[55.6–124.8]	142.1	[67.7–287.7]
Normal spermatozoa (%)*	29	[17–42]	38	[26–44]
MAI	1.87	[1.65–2.06]	1.86	[1.68–1.94]

*P < .05 between cases and controls. *MAI* multiple anomalies index

### DNA fragmentation and chromatin condensation (Fig. 2)

No statistical difference between the URPL group and controls was found for DFI (cases 13.15% [7.95–20.85] vs controls 13.95% [11.85–19.40]), HDS (4.20% [3.45–5.20] vs 4.60% [3.85–5.20]) or TUNEL (5.30% [2.00–8.47] vs 5.20% [3.30–7.75]). There was no significant difference in aniline blue staining between the two groups (6% [3.5–14] for the URPL group vs 9% [2–18] for controls).

**Fig. 2:**
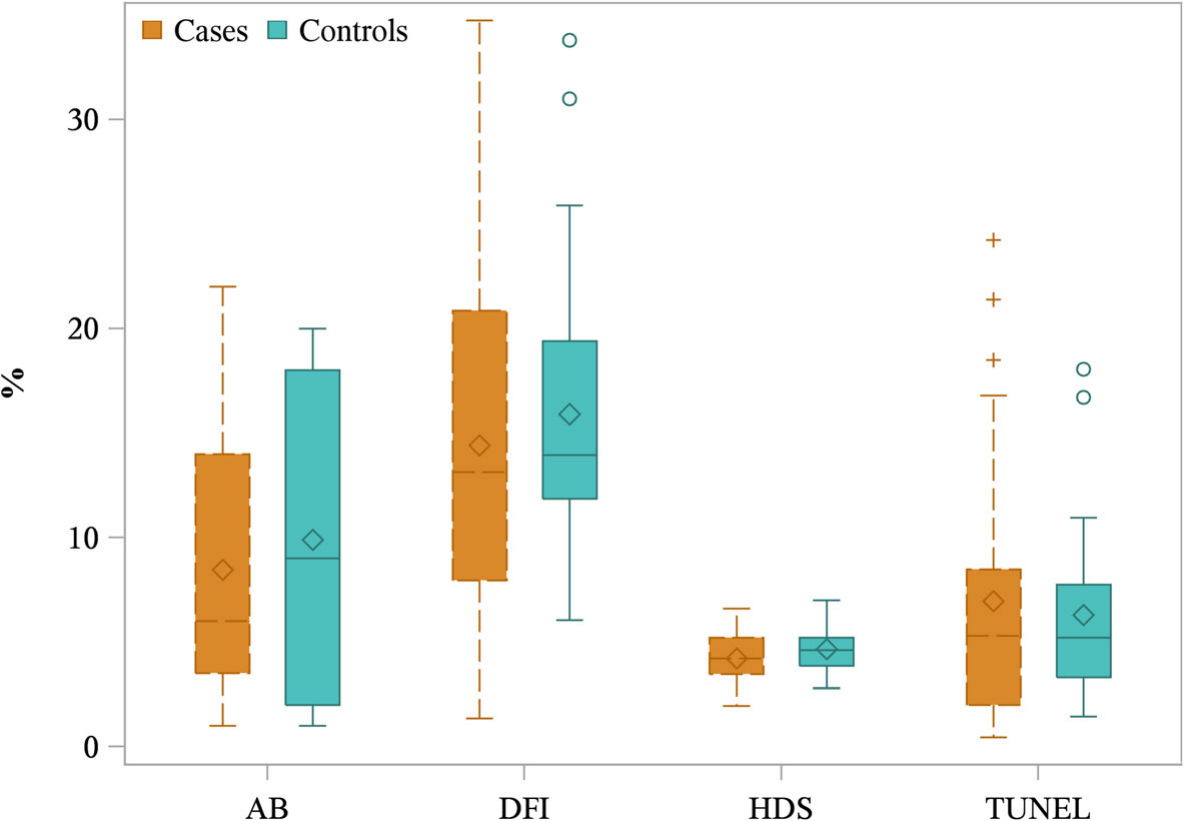
Evaluation of sperm nuclei in men with URPL and controls. Data are presented as median [q1–q3] boxplot (median is represented as the line under the boxplot, the mean as the diamond, q1 and q3 as the border of the box, and circles represent the outliers). AB, aniline blue staining; DFI, DNA fragmentation index; HDS, high DNA stainability; TUNEL, terminal deoxynucleotidyl transferase dUTP nick end labeling

### Sperm aneuploidy (Table 3 and Fig. 3)

A mean of 5264 ± 206 cells per subject were read for the URPL group (a total of 168,431 spermatozoa) and 5240 ± 190 for the control group (a total of 141,461 spermatozoa).

**Table 3:** Aneuploidy Analysis of the Spermatozoa of the 33 Cases and 27 Controls

	Cases		Controls	
	median	[q1–q3]	median	[q1–q3]
Y18	49.48	[48.64–50.26]	49.43	[47.67–50.60]
X18	49.62	[49.02–50.37]	49.82	[48.85–51.30]
XX18	0.04	[0.02–0.06]	0.06	[0.04–0.06]
YY18	0.02	[0.02–0.04]	0.04	[0–0.06]
XY18*	0.52	[0.41–0.81]	0.21	[0.13–0.31]
X1818*	0.04	[0–0.04]	0.00	[0–0.02]
Y1818	0.04	[0–0.08]	0.02	[0–0.04]
X1818 + Y1818*	0.08	[0.04–0.10]	0.04	[0–0.06]
XY1818	0	[0–0]	0	[0–0]
XX1818	0	[0–0]	0	[0–0]
YY1818	0	[0–0]	0	[0–0]
XXY18	0	[0–0]	0	[0–0]
18	0.28	[0.17–0.44]	0.24	[0.17–0.33]
X*	0	[0–0.07]	0.06	[0–0.23]
Y	0	[0–0]	0	[0–0]
Total aneuploidy**	1.07	[0.82–1.32]	0.65	[0.46–0.81]

Aneuploidy values are given as percentages

*p < .05 between cases and controls

**p < .001

**Fig. 3:**
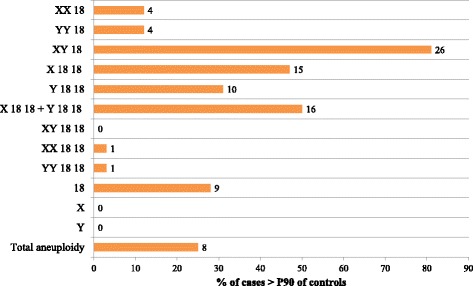
Percentage of men with URPL who had increased aneuploidy compared with the 90th percentile (P90) of controls. Columns represent the percentage of cases, numbers in the figure are the number of cases for each category

The rates of normal haploid X18 or Y18 spermatozoa did not differ between the URPL group and the control group (Table 3, X18: 49.62% [49.02–50.37] for URPL vs 49.82% [48.85–51.30] for controls; Y18: 49.48% [48.64–50.26] vs 49.43% [47.67–50.60]). The number of spermatozoa that were disomic and nullisomic for sex chromosomes also did not significantly differ between the two groups. The chromosome 18 disomy rate was twice as high in the URPL group than in the control group (0.08% [0.04–0.10] vs 0.04% [0–0.06] respectively, *P* = .003). We also found a significant increase (*P* < .001) in the number of hyperhaploid cells in the URPL group (0.52% [0.41–0.81]), which was more than twice as high as in the control group (0.21% [0.13–0.31]). Overall, total aneuploidy was significantly higher in the URPL group than in controls (1.07% [0.82–1.32] vs 0.65% [0.46–0.81], *P* < .001).

When compared with the 90th percentile (P90) of controls, 26 cases (79%) presented an increased number of hyperhaploid spermatozoa, 16 cases (49%) showed an increase in chromosome 18 disomy and 8 (24%) cases showed an overall increased aneuploidy rate (Fig. 3).

**Fig. 4:**
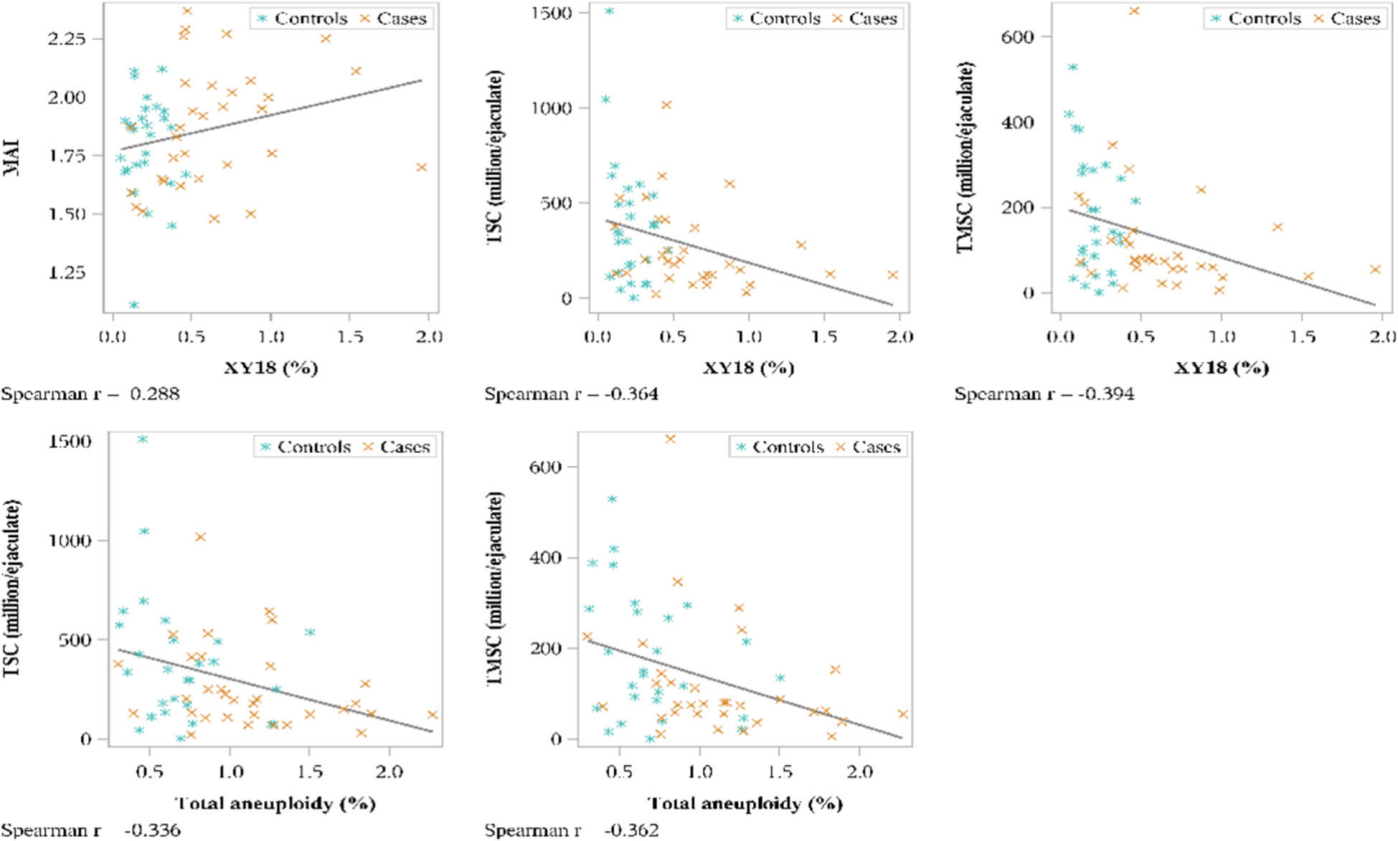
Correlation between aneuploidy, sperm parameters and morphology for men with URPL and controls. TSC, total sperm count; TMSC, total motile sperm count; MAI, multiple anomalies index

There was a significant positive correlation between the multiple anomalies index (MAI) and hyperhaploid cells (*P* = .027), and negative correlations between total sperm count and total aneuploidy (*P* = .009), total motile sperm count and total aneuploidy (*P* = .004), total sperm count and hyperhaploid cells (*P* = .004), and total motile sperm count and hyperhaploid cells (*P* = .002) (Fig. 4). We found no correlation between fragmentation and hyperhaploidy, fragmentation and chromosome 18 disomy, or fragmentation and total aneuploidy in either cases or controls (data not shown).

## Discussion

To our knowledge, our study is the only one that includes an extensive andrological examination and a questionnaire about family history, living environment, lifestyle and exposure. Our prospective case-control study, including 33 cases of men from couples experiencing URPL and 27 controls of proven fertility, analyzed semen characteristics, sperm morphology, sperm DNA status and sperm aneuploidy. To date, only one other study has performed the same type of analyses, but in a smaller population [7]. All other research has focused either on sperm characteristics, morphology and DNA status [2, 4, 6, 8–10, 13–15, 21–26], on sperm characteristics, morphology and aneuploidy [3, 5, 11, 17], or on DNA status and aneuploidy [16].

Our results highlight a significant difference in family history of infertility and miscarriage in couples experiencing URPL. This could suggest the implication of a potential genetic factor. However, patient bias is also a possibility, as the cases are probably more aware of issues related to miscarriages and infertility than the control group and have a better knowledge of family infertility and miscarriage history. Furthermore, the questionnaire revealed a significant difference in the living environment between controls and cases. Controls predominantly lived in large urban areas, whereas cases more frequently lived in small urban areas. People living in the country may be more prone to exposure to various factors such as pesticides that could have an impact on pregnancy loss, as it has been shown that exposure to some types of pesticides was associated with early abortions [27]. However, this cannot be confirmed as we can also argue that the controls were enrolled in three large urban maternity departments where the population is mainly composed of people living in the city or close suburbs, while the cases more frequently came from the whole region, including close suburbs and more rural areas.

We found that TMSC was significantly lower in men from the URPL group than in men from the control group. This finding is in agreement with previously published results [2, 3, 6–9], with the exception of two studies [5, 28]. Battacharya et al. suggested that motility alteration was related to damaged plasma membrane which can affect the sperm’s DNA integrity to an extent where although the oocyte can be fertilized, the embryo later fails to grow correctly, leading to a miscarriage [2]. In the same field, Gil-Villa et al found increased levels of oxidant components in the semen of men in an URPL group, and these possibly affect the cellular components necessary for correct embryo development and sperm parameters such as motility [9]. Kazerooni et al found that abnormal sperm parameters in men in a URPL group were correlated with protamine deficiency, which is known to affect normal embryo development [8]. These three studies seem to link, though indirectly, impaired motility to impaired embryo development and miscarriage. In conclusion, we do not know if impaired motility is directly involved in miscarriage, but it could reflect sperm dysfunction in these men that could lead to URPL.

We observed a significant increase in chromosome 18 disomy, hyperhaploidy and total aneuploidy in the men from the URPL group compared with the fertile controls. This confirms previously published data that show a trend to increased chromosome 18 disomy in men in an URPL group [3]. Other studies on URPL and the search for chromosomal abnormalities have found increased incidence of sperm aneuploidy [7, 11, 16, 17]. Collodel et al. found increased diploidy, disomy and total aneuploidy in 50% of their cases when comparing the median and 75th percentile with their controls [5]. In our study, we found that sex chromosome sperm nondisjunction (XY18) and 18 disomy values were above the 90th percentiles of the control group in 79% and 49% of cases, respectively. These data all seem to indicate that increased anomalies in chromosome numbers have an impact on URPL, possibly involving a male factor, as has already been suggested in pregnancy losses after intracytoplasmic sperm injection (ICSI) [25, 29] as well as in implantation failure [25, 29]. Regarding DNA fragmentation and URPL, there seems to be a discrepancy in published results: some show a tendency to an increased fragmentation in cases of URPL [2, 4, 6–8, 10, 13–15, 21–24, 28] whilst others report no differences between cases and controls [9]. In our study we found no significant difference in DNA fragmentation between cases and controls, using either SCSA or TUNEL.

One limitation of our study is the small number of chromosomes studied by FISH (X, Y, 18). However, an interchromosomal effect has been reported using array-comparative genomic hybridization (CGH) [30, 31] and it has been suggested that the results obtained by studying one set of chromosomes could be extended to others [32]. While this study is one of the largest concerning the number of participants, further extensive population studies are required to investigate whether environmental factors may be associated with URPL.

## Conclusions

To conclude, the increased aneuploidy rate in spermatozoa could explain URPL. In ICSI, selection of euploid spermatozoa could possibly improve the chances of these couples of successfully carrying a pregnancy to term. However, as yet no such technique is available.

Preimplantation genetic screening (PGS) could also be useful in managing URPL, especially as our results indicate that spermatozoa in these patients show an increased aneuploidy that could lead to an aneuploid embryo. Screening genetically normal embryos could improve chances of successful implantation and pregnancy. But PGS in cases of URPL is controversial. One study showed that in cases of increased sperm aneuploidy PGS seemed to prevent miscarriages and increased the chances of a successful pregnancy [33], whereas another more recent study showed that PGS did not improve the chances of a successful pregnancy compared with expectant management [34]. However, in this latter study, sperm aneuploidy status was not investigated. In view of our results and those of Rubio et al., study of sperm chromosome status in men whose partners experience URPL must be continued. PGS may be a useful option in the management of URPL when sperm are found to be aneuploid, but further studies on the results of PGS in this context should be done to confirm this hypothesis.

## Abbreviations

ART: Assisted reproduction techniques
BMI: Body mass index
DFI: DNA fragmentation index
FISH: Fluorescence in situ hybridization
HDS: High DNA stainability
ICSI: Intracytoplasmic sperm injection
MAI: Multiple anomalies index
PGS: Preimplantation genetic screening
RPL: Recurrent pregnancy loss
SC: Sperm count
SCSA: Sperm chromatin structure assay
TMSC: Total motile sperm count
TSC: Total sperm count
TUNEL: Terminal deoxynucleotidyl transferase dUTP nick end labelling
URPL: Unexplained recurrent pregnancy loss
